# The FCTC Turns 10: Lessons From the Fist Decade

**DOI:** 10.2188/jea.JE20160080

**Published:** 2016-06-05

**Authors:** Heather Wipfli

**Affiliations:** 1 Department of Preventative Medicine, Keck School of Medicine, University of Southern California, Los Angeles, CA, USA; 2 University of Southern California Institute for Global Health, Los Angeles, CA, USA

**Keywords:** tobacco control, Framework Convention on Tobacco Control, Global Health Governance

## Abstract

The Framework Convention on Tobacco Control (FCTC) stands as a landmark approach to addressing a global health problem. It represents the first time the World Health Organization (WHO) used its constitutional right to negotiate an international law and the first time the Member States of WHO agreed to a collective response to chronic, non-communicable diseases. This paper draws lessons from the FCTC’s first decade in force and explores what aspects of the FCTC experience can inform future efforts to address other disease epidemics driven by corporate activity, such as alcohol and food.

The Framework Convention on Tobacco Control^1^ (FCTC) stands as a landmark approach to addressing a global health problem. It represents the first time the World Health Organization (WHO) used its constitutional right to negotiate an international law and the first time the Member States of WHO agreed to a collective response to chronic, non-communicable diseases. Originally adopted by the World Health Assembly in 2003, the treaty has now been binding international law for over a decade, making this a good time to reflect back on lessons learned from public health’s first foray into international law making. These lessons can inform future efforts to address other disease epidemics driven by corporate activity, such as alcohol and food.

One of the first lessons that we can draw from the FCTC process was the need for a clear evidence on the target for control—in this case cigarettes produced by large transnational tobacco companies. The scientific conclusion that cigarette smoking causes disease is often dated back to the release of the 1964 Surgeon General’s Report on Smoking and Health,^2^ which concluded that smoking caused lung cancer in men, and recommended regulatory action to control consumption. Since 1964 the US Surgeon General has published over three dozen reports linking active and passive smoking to a large number of cancers and other diseases in adults and children. Other countries have replicated the model of consolidating the accumulated evidence followed by calling for action, including the Tobacco Free Japan report^3^ and the report from the Chinese Health Ministry, entitled China Report on the Health Hazards of Smoking.^4^

Despite the plethora of evidence on tobacco and disease, global efforts to control tobacco were largely non-existent in the 20th century leading the renown epidemiologist Sir Richard Doll to exclaim “That so many diseases—major and minor—should be related to smoking is one of the most astonishing findings of medical research in this century…less astonishing perhaps than the fact that so many people have ignored it.”^5^ Consequently, the second lesson we can draw from the FCTC experience is that health effects alone do not generate action; the scope of the burden of disease is also critical. In the case of tobacco, over four million deaths were attributable to tobacco at the start of the FCTC process in 1998 and now (in 2016) over five million deaths per year are attributable to tobacco. It was and remains the largest cause of preventable death in the world and is projected to kill over one billion people in the 21st century. The global tobacco epidemic also disproportionately impacts low- and middle-income countries (LMIC) with over 70% of tobacco deaths projected to occur in LMICs by 2020. Tobacco’s negative impact on individual and national economic development has been increasingly recognized, and is identified with the in the 2015 Sustainable Development Goals.^6^

Still, evidence of individual and population harm is not enough to base an international regime around; specific, evidence-based control measures are needed. In the case of tobacco, we had over a half-decade of experience in reducing tobacco use in diverse high-income countries, as well as a handful of middle-income countries. The measures that were found most effective include taxation (the most effective), health warning labels, advertising bans, and secondhand smoke bans (Figure 1). These policies provided the basic framework around which to build an international legal regime for tobacco and the final FCTC text includes articles on each of these measures.^1^

**Figure 1.**
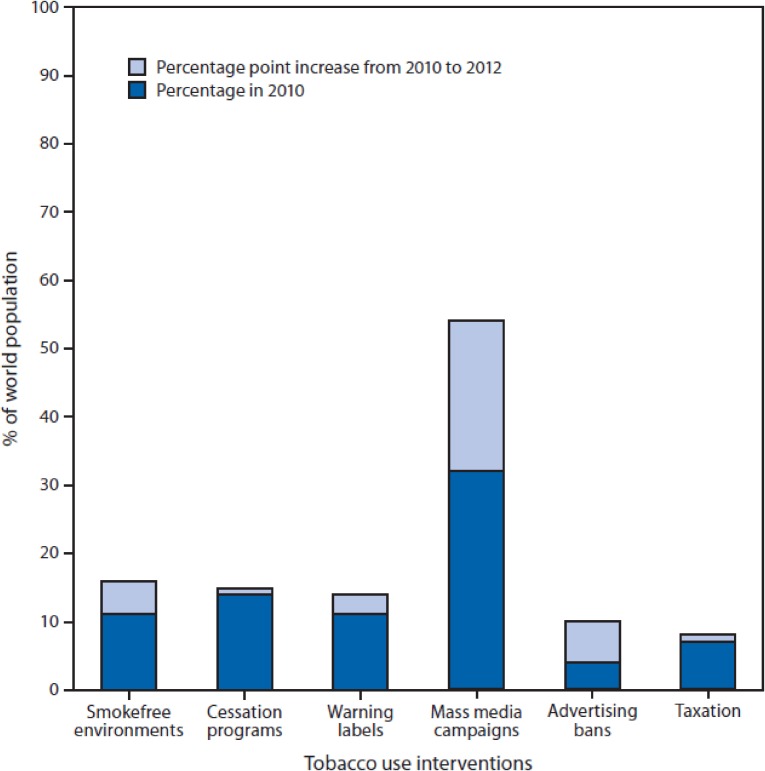
Adult* per capita cigarette consumption and major smoking and health events, United States, 1990–2012 *Adults ≥ 18 years of age as reported annually by the Census Bureau Source: U.S. Department of Health and Human Services, National Institutes of Health, & National Cancer Institute. (2014). *The Health Consequences of Smoking 50 Years of Progress: A Report of the Surgeon General*. Atlanta, GA: U.S. p.18.

In a rational world, causal evidence of disease and death in individuals and populations, and a suite of proven control measures would lead to quick decisive action to protect health. However, in the case of tobacco and other diseases driven by corporate activity, it became crucial to know and challenge industry activities. The global tobacco industry is highly concentrated. There are only five major companies—Phillip Morris, British American Tobacco (BAT), Japan Tobacco International and China National Tobacco Company, a state monopoly. In some ways, this concentration of the industry makes it easier to identify and control, although the company size and wealth pose significant challenges, especially when trying to advance tobacco control in lower-income countries (Figure 2). Since the mid-1980s, in part due to successful tobacco control campaigns in high-income countries, the tobacco industry has increasingly targeted low- and middle-income markets with their products. Consequently, an increasingly percentage of their wealth is derived from these markets. The vast wealth of transnational tobacco companies dwarfs many low- and middle-income economies, making it even more difficult to undertake control efforts that could undermine foreign direct investment opportunities or other economic benefits generated from industry pressures.

**Figure 2.**
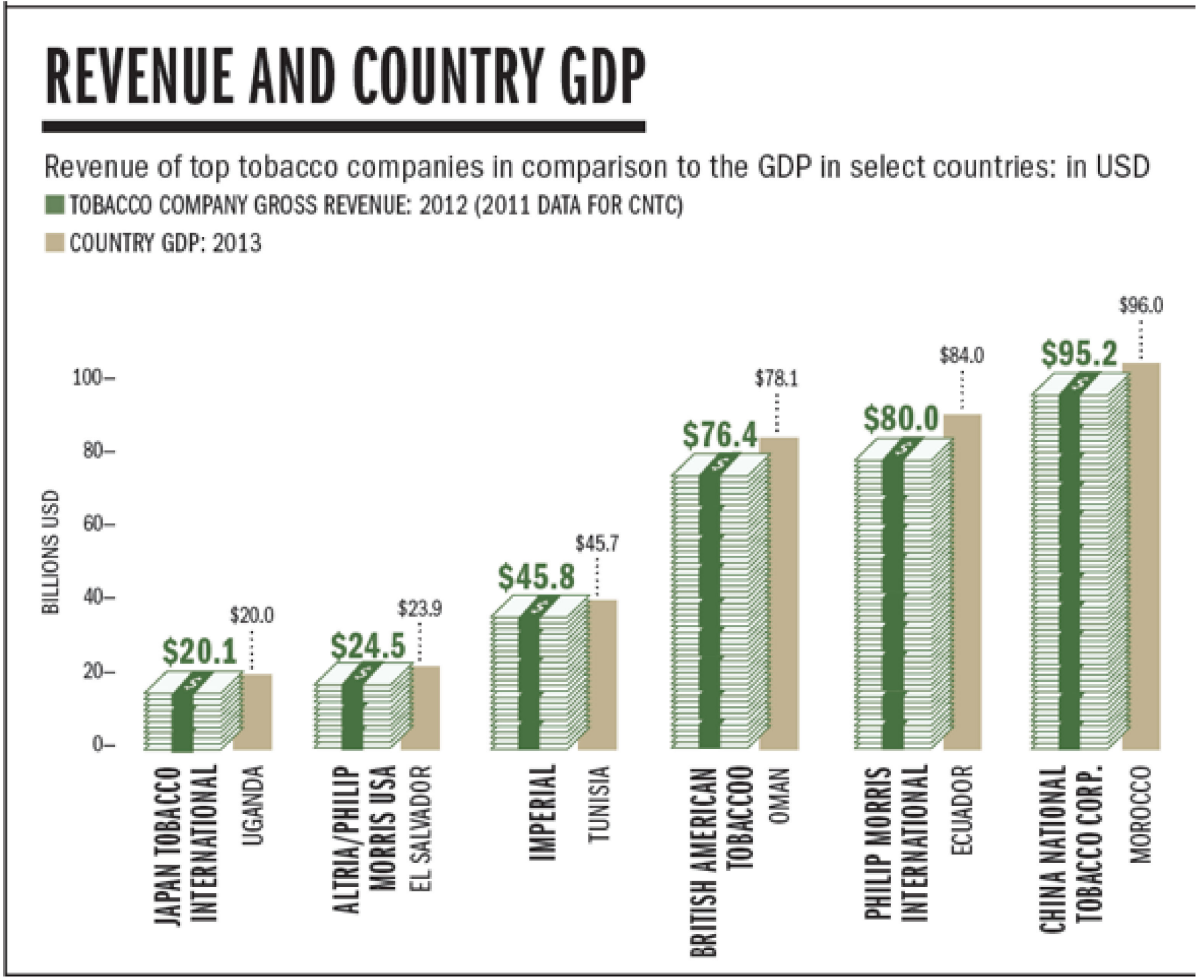
Revenue of top tobacco companies in comparison to the GDP in select countries: in USD Source: Eriksen M, Mackay J, Schluger N, Gomeshtapeh FI, & Drope J. (2015). *The Tobacco Atlas, 5th Edition*. Retrieved from Atlanta: www.tobaccoatlas.org. p48.

The tobacco industry has used numerous strategies to expand their markets and avoid regulation. The release of millions of previously confidential industry documents in 1998 as a result of litigation in the U.S. uncovered a treasure trove of information regarding the industry’s decades-long campaign to deceive the public about the effects of tobacco on health, and their targeted efforts to recruit consumers in vulnerable populations such as youth and minorities. The release of documents corresponded with the launch of the FCTC, and WHO decided to undertake a study of the documents as they pertained to the industry’s approach to WHO. The report found that the industry viewed WHO as a “leading enemy”^7^ and had paid consultants to “attack WHO”^7^ and “discredit key individuals”.^7^ A key strategy was to “contain, neutralize, reorient WHO”^7^ away from tobacco control and other non-communicable diseases. As a result of this report, WHO declared that the industry could not be seen as a credible stakeholder in the FCTC process and banned them from participating in the negotiations, even as observers. The only time the industry was invited to participate was during the FCTC public hearing held just prior to the first working group session for the FCTC. The hearings provided the first global forum in which tobacco companies acknowledged the deadly effects of active smoking. Still, however, some companies such as Japan Tobacco International, continued to dispute the scientific evidence that secondhand smoke causes disease.^8^

The need to combat industry influence was widely discussed throughout the FCTC negotiations and Article 5.3 of the final FCTC text requires that “all Parties shall act to protect these policies [with respect to tobacco control] from commercial and other vested interests of the tobacco industry in accordance with national law”.^1^ However, keeping the industry at bay requires more than policies on paper. It requires surveillance and strong advocacy on the part of both governmental and non-governmental stakeholders. There are a number of noteworthy individuals without whose advocacy the FCTC would never have existed: Ruth Roemer, a public health professor in California, championed the idea of the FCTC from a vague theory to a practical option; Judith MacKay in Hong Kong, stood up to the seemly impenetrable tobacco industry in China and advocated global leaders at WHO and beyond to take action to control tobacco; Gro Harlem Brundtland, the Director General of WHO, launched the innovative FCTC development process, and fought tobacco industry challenges; and team member Derek Yach, who lead the WHO secretariat through the early days of FCTC negotiations. At the national level there were also leaders, such as Gong Huang Tang in China, and Yumiko Mochizuki in Japan, who worked for decades collecting the data and presenting evidence to support control efforts in their respective countries.

Non-governmental organizations (NGOs) were essential in supporting FCTC negotiations and working to ensure strong implementation of the treaty. NGOs have been particularly good at ‘shaming and blaming’ countries that fail to live up to public health standards. During FCTC negotiations, the Framework Convention Alliance, a network of hundreds of NGOS from throughout the world, released a daily bulletin identifying the best (the Orchid Award) and the worst (the Dirty Ashtray Award) interventions from the previous day. Countries were surprisingly sensitive to being called out for being unsupportive of public health. NGOs also served a key role in educating country delegates about FCTC and helping draft strong public health text. In the decade since FCTC was entered into force NGOs, many supported by the massive influx of funding provided by former New York City mayor, Michael Bloomberg, have served as key capacity builders and monitors of the treaty at the country level.

As just mentioned, in 2006 Michael Bloomberg launched a global initiative supporting FCTC implementation. Starting with an initial $USD500 million grant, the Bloomberg Initiative has now committed over $USD650 million to global tobacco control efforts. This funding has been critical in implementing the FCTC during its first decade. However, the massive Bloomberg grant is not, and was not meant to be, sustainable. Countries must develop national capabilities to fund their national tobacco control programs over the long term. Earmarked tobacco taxes provide one effective approach to raising money for tobacco control, while at the same time promoting tobacco cessation. At the recent United Nations Financing Sustainable Development Summit in Addis Ababa, tobacco taxation was recognized as a powerful tool for raising funds to support public health and development. Together, the health evidence, global surveillance, proven control measures, advocacy and funding resulted in the successful negotiation and implementation of the FCTC. Policy changes between 2007–2010 are estimated to result in 7.5 million fewer smoking-related deaths by 2050. Nearly 1.3 billion lives were newly protected by at least one FCTC measure between 2008–2013, and 900 million additional people were protected by smoke-free bans between 2007–2012 (Figure 3). Still the battle is not over. The industry continues to attack using novel control strategies, such as plain packaging in Australia, through trade treaties and intellectual property agreements. The industry is using new forms of social media to avoid advertising bans and to recruit new users. The industry is continually diversifying its product offerings, as seen with the rapid increase in the use of electronic cigarettes. Our ongoing challenge is signified by tobacco industry stock prices, which more than doubled between 2009 and 2014, and which continue to climb.

**Figure 3.**
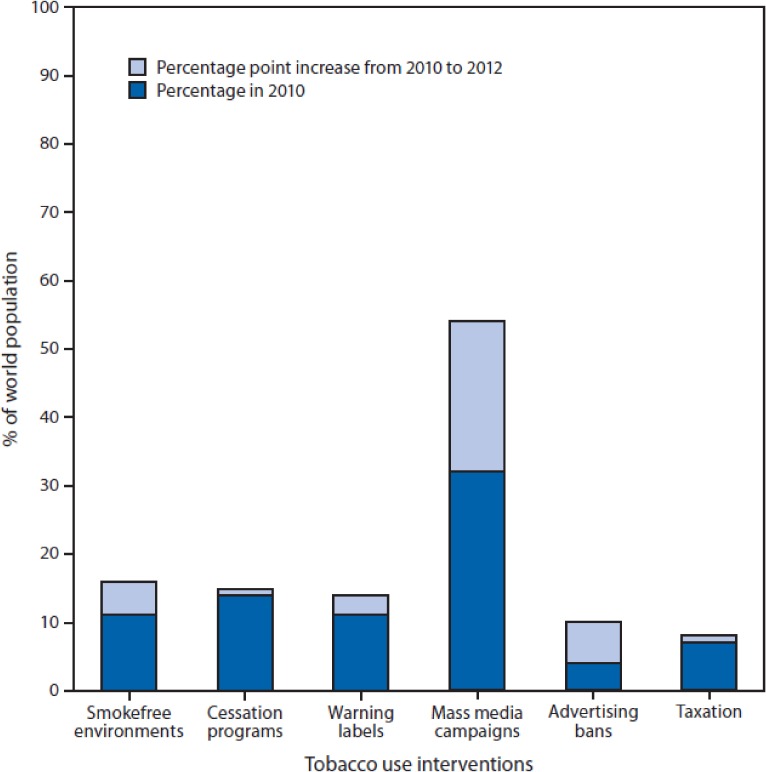
Percentage of the world population covered by MPOWER interventions against tobacco use, 2010 and 2012 Source: Centers for Disease Control and Prevention. (2014). CDC Grand Rounds: Global Tobacco Control. *Morbidity and Mortality Weekly Report (MMWR).* Retrieved from http://www.cdc.gov/mmwr/preview/mmwrhtml/mm6313a1.htm. Source: World Health Organization. WHO report on the global tobacco epidemic 2013: enforcing bans on tobacco advertising, promotion, and sponsorship. Geneva, Switzerland: World Health Organization; 2013. Available at http://www.who.int/tobacco/global_report/2013/en. Alternate Text: The figure above is a stacked bar chart that shows the percentage of the world population covered by MPOWER interventions against tobacco use in 2010 and the percentage-point increase from 2010 to 2012.
